# KPC1 alleviates hypoxia/reoxygenation‐induced apoptosis in rat cardiomyocyte cells though BAX degradation

**DOI:** 10.1002/jcp.28854

**Published:** 2019-05-30

**Authors:** Ye Yuan, Yong‐yi Wang, Xin Liu, Bin Luo, Lei Zhang, Fei Zheng, Xing‐Yuan Li, Ling‐Yun Guo, Lu Wang, Miao Jiang, Ya‐mu Pan, Yu‐wen Yan, Jian‐ye Yang, Shi‐You Chen, Jia‐Ning Wang, Jun‐Ming Tang

**Affiliations:** ^1^ Institute of Clinical Medicine and Department of Cardiology, Renmin Hospital, Hubei University of Medicine Shiyan Hubei China; ^2^ Department of Cardiovascular Surgery, Ren Ji Hospital, School of Medicine Shanghai Jiao Tong University Shanghai China; ^3^ Department of Physiology, School of Basic Medicine Science Hubei University of Medicine Hubei China; ^4^ Institute of Biomedicine and Key Lab of Human Embryonic Stem Cell of Hubei Province Hubei University of Medicine Hubei China; ^5^ Laboratory Animal Center Hubei China; ^6^ Department of Physiology & Pharmacology The University of Georgia Athens USA

**Keywords:** apoptosis, Bax, cardiomyocyte, KPC1, mitochondrial function

## Abstract

Bax triggers cell apoptosis by permeabilizing the outer mitochondrial membrane, leading to membrane potential loss and cytochrome *c* release. However, it is unclear if proteasomal degradation of Bax is involved in the apoptotic process, especially in heart ischemia‐reperfusion (I/R)‐induced injury. In the present study, KPC1 expression was heightened in left ventricular cardiomyocytes of patients with coronary heart disease (CHD), in I/R‐myocardium in vivo and in hypoxia and reoxygenation (H/R)‐induced cardiomyocytes in vitro. Overexpression of KPC1 reduced infarction size and cell apoptosis in I/R rat hearts. Similarly, the forced expression of KPC1 restored mitochondrial membrane potential (MMP) and cytochrome *c* release driven by H/R in H9c2 cells, whereas reducing cell apoptosis, and knockdown of KPC1 by short‐hairpin RNA (shRNA) deteriorated cell apoptosis induced by H/R. Mechanistically, forced expression of KPC1 promoted Bax protein degradation, which was abolished by proteasome inhibitor MG132, suggesting that KPC1 promoted proteasomal degradation of Bax. Furthermore, KPC1 prevented basal and apoptotic stress‐induced Bax translocation to mitochondria. Bax can be a novel target for the antiapoptotic effects of KPC1 on I/R‐induced cardiomyocyte apoptosis and render mechanistic penetration into at least a subset of the mitochondrial effects of KPC1.

## 1 INTRODUCTION

Coronary heart disease (CHD), known for acute myocardial infarction (MI), is the mainspring of mortality and morbidity worldwide. Clinically, both thrombolytic therapy and percutaneous coronary intervention (PCI) have routinely been applied to restore myocardial perfusion of MI (Fokkema, Vogelzang, Vlaar, & Zijlstra, 2009). Although this treatment is timely and effective for mitigating acute MI injury and limiting MI size after reperfusion, the process of reperfusion can itself drive cardiomyocyte apoptosis or death, which is known as myocardial ischemia‐reperfusion (I/R) injury (Ong, Samangouei, Kalkhoran, & Hausenloy, 2015). Accumulated data have shown that several typical factors, including oxidative stress, Ca^2+^ overload, and mitochondria dysfunction, synergistically mediate the infaust effects of myocardial I/R injury (Lesnefsky, Chen, Tandler, & Hoppel, 2017). However, the specific therapeutic modality has not still been effectively established.

Proteasomal functional insufficiency (PFI) or lysosomal insufficiency has been reported as a major cardiac etiological element (Wang & Robbins, 2013). Indeed, PFI induced myocardial ubiquitinated proteins accumulation have been observed in nearly all heart disease animal models, such as MI (Day et al., 2013), myocardial I/R (Tian et al., 2012), pressure‐overloaded hypertrophy (Ranek et al., 2015), and desmin‐related cardiomyopathy (DRC; Wang, Su, & Ranek, 2008). Furthermore, the proteasome inhibitor, bortezomib, has adverse cardiac effects in multiple myeloma patients, including severe heart failure, have been reported (Bockorny, Chakravarty, Schulman, Bockorny, & Bona, 2012; Gupta, Pandey, & Sethi, 2012). These evidence show that PFI plays a role in cardiac pathogenesis (Pagan, Seto, Pagano, & Cittadini, 2013). Therefore, enhancing proteasomal function in pathological conditions will be a hopeful strategy for patients with PFI.

Actually, data from bench to bedside have identified cardiomyocyte apoptosis as a typical event in myocardial I/R (Zhang et al., 2013). Further evidence has suggested that the permeabilization of the mitochondrial outer membrane by Bax during apoptosis is considered a key step and a point of no return in the signaling pathway, mainly attributed to the altered traits in Bax expression and mitochondria translocation (Gross, Jockel, Wei, & Korsmeyer, 1998). Previous study found that proteasomal degradation of Bax involved in the fine tuning of apoptotic cell death (Dewson, Snowden, Almond, Dyer, & Cohen, 2003). And, a novel mechanism which the proapoptotic activity of Bax was inhibited by ubiquitination has been reported (Cakir et al., 2017). Of interest, KPC1, a novel E3 ubiquitin ligase, could mediate ubiquitination and proteasomal processing of NF‐κB1 and p27Kip1, and play a crucial role in cell growth (Kamura et al., 2004; Kravtsova‐Ivantsiv & Ciechanover, 2015; Kravtsova‐Ivantsiv et al., 2015; Lu et al., 2009). The present study, for the first time, has shown that KPC1 binds to Bax, and controls Bax stability and mitochondria translocation in hypoxia and reoxygenation (H/R) H9c2 cells, providing a novel therapeutic modality for I/R through reducing Bax mitochondria translocation whereas accelerating proteasomal degradation of Bax.

## 2 MATERIALS AND METHODS

**Animals.** All animals were served in accordance with the Guide for the Care and Use of Laboratory Animals. All operation procedures for I/R model of hearts in rats were permitted by the Committee of Experimental Animals Care of Hubei University of Medicine.

**Human samples.** The study was performed strictly following international ethical guidelines for biomedical research involving human subjects published by CIOMS and was approved by the Institutional Review Board of Shiyan Renmin Hospital, Hubei University of Medicine. Human hearts specimens were collected during the post mortem examination with informed consent from patients or family members. Written informed consent was obtained from all participating individuals.

### 2.1 Adenovirus pretreatment and ischemia‐reperfusion rat model

Male SD rats (250–300 g) provided by the Laboratory Animal Center (Hubei University of Medicine) were randomized into four groups: an Ad‐green fluorescent protein (GFP) pretreatment sham group, an Ad‐KPC1 pretreatment sham group, an Ad‐GFP pretreatment and ischemia‐reperfusion (I/R) group, and an Ad‐KPC1 pretreatment and I/R group. After anesthetized with chloral hydrate (300 mg/kg) by intraperitoneal injection, Ad‐GFP or Ad‐KPC1 (1.0 × 10^9^ pfu in 150 μl) was injected at three different points (50 μl per site) into myocardium sites in the left ventricle anterior wall. Seven days later, ischemia‐reperfusion was achieved by the ligature of the left coronary artery anterior descending branch (LAD) as described previously (Tang, Xie, Pan, Wang, & Wang, 2006). In brief, after anesthetized, the rats were ventilated with a rodent ventilator (HX‐300S, Techman Soft) and then the LAD was ligated for 1 hr followed by a 24 hr reperfusion by thoracotomy surgery of the fourth intercostal space.

### 2.2 TTC staining

After a 24 hr reperfusion, the rats were narcotized and the hearts were quickly removed. After frozen at −20°C for 30 min, the hearts were transverse section into four pieces with 2–3 mm thickness. The sections were incubated in 1% TTC (2, 3, 5‐triphenyltetrazolium chloride) at 37°C for 20 min lucifugally in accordance with the instructions of manual (D025, Nanjing Jiancheng Bioengineering Institute). The arithmetic of infarcted zone volume was described as previously (Huang et al., 2011).

### 2.3 Heart tissues TUNEL assay

After fixed with 4% paraformaldehyde and embedded in paraffin, the hearts were serially cut for obtaining sections. For detecting apoptosis, a Colorimetric terminal‐deoxynucleotidyl transferase‐mediated nick end labeling (TUNEL) Apoptosis Assay Kit (C1091, Beyotime) was used. The number of apoptotic cells was counted using a high‐power light microscope when reddish brown cells were observed under adjacent six fields. Apoptotic ratio was calculated by the number of positive cells/total number of cells × 100%.

### 2.4 Cell culture and hypoxia reperfusion treatment

H9c2 cells were cultured in Dulbecco's Modified Eagle's Medium (DMEM; Invitrogen) supplemented with 15% fetal bovine serum (FBS; Gibico) at 37°C under 95% air and 5% CO_2_. The cells were transfected with adenovirus (5 × 10^9^ pfu) expressing KPC1 short‐hairpin RNA (shRNA) or overexpressing KPC1 at 70–80% confluence. After 24 hr, cells were exposed under 90% N_2_, 5% O_2_, and 5% CO_2_ at 37°C for 24 hr in DMEM. Afterwards, the cells were cultured in DMEM supplemented with 15% FBS under normoxia for 3 hr and then were used in the subsequent experiments (Huang et al., 2011).

### 2.5 Construction of KPC1 short‐hairpin RNA (shRNA) and overexpression adenoviral vector

The KPC1 shRNA sequences are (5′–3′): ACGCGTGAGTTCCTGCTTAGCAATGTCCTTCAAGAGAGGACATTGCTAAGCAGGAACTTTTTTTGGAAA (sense) and AAGCTTTTTCCAAAAAAAGTTCCTGCTTAGCAATGTCCTCTCTTGAAGGACATTGCTAAGCAGGAACTCAC (antisense). The adenovirus vector was constructed, packed and purified (Zhang et al., 2013).

### 2.6 Western blot analysis

The cells were lysed with radioimmunoprecipitation assay (RIPA) (Millipore) buffer and quantified with a bicinchoninic acid (BCA) Protein Assay Kit (23225, Thermo Fisher Scientific). After sodium dodecyl sulfate‐polyacrylamide gel electrophoresis (SDS‐PAGE), samples were electro‐transferred onto polyvinylidene difluoride (PVDF) membranes (BioRad). After incubated with primary antibodies (anti‐Bax, #2772, CST; anti‐KPC1, ab57549, abcam) and horseradish peroxidase (HRP)‐conjugated secondary antibodies, the membranes were visualized with enhanced chemiluminescence (ECL) reagent (BioRad).

### 2.7 H9c2 cells TUNEL assay

Forty‐eight hours after treated with Ad‐KPC1, Ad‐Ctrl, Ad‐shKPC1, or Ad‐shCtrl, the cells were exposed to specific treatment with H/R. H9c2 cell apoptosis was measured with an In Situ Cell Death Detection Kit (#12156792910, Roche). Finally, the positive cells from the TUNEL assay were directly analyzed with fluorescence microscope after staining the nucleus with 4',6‐diamidino‐2‐phenylindole (DAPI). The ratio of TUNEL‐positive cells was evaluated as TUNEL‐positive cells per high field/total cells per high field (Zhao et al., 2013).

### 2.8 Immunofluorescence staining

The cultured H9c2 cells in 24‐well plates reached 70% cell density. After H/R treatment, 4% polyoxymethylene was used to fix the cells for 15 min, which were washed three times. After blocking the nonspecific antigen, the primary antibodies, such as Bax (#2772, CST), cytochrome *c* (sc‐13156, Santa Cruz), KPC1 (ab57549, abcam), and Troponin T‐C (cTnT, sc‐515899, Santa Cruz) were added. The specific well with the corresponding second antibody (1:250) added was incubated 2 hr at room temperature. Five random field of each glass slide (Thermo Fisher Scientific) were photographed and total 30 images per group were obtained according to the same standard. Images were analyzed by three technicians who did not know grouping information using ImageJ (Java) software (National Institutes of Health).

### 2.9 Immunohistochemistry (IHC) staining

The hearts were fixed in 4% paraformaldehyde and embedded in paraffin. 4 µm thickness sections were rehydrated, blocked and then incubated with primary antibodies: rabbit anti‐Bax (1:100, #2772, CST) and mouse anti‐KPC1 (1:100, ab57549, abcam). Then, the sections were incubated with secondary antibodies followed by counterstaining with hematoxylin.

### 2.10 Flow cytometry assay

To quantitatively analyze the role of KPC1 overexpression in H9c2 cell apoptosis, 48 hr after transfected with Ad‐KPC1 or Ad‐Ctrl, the cells were exposed to specific treatment with H/R. Because Ad‐KPC1 did not carry the specific GFP‐tag, an Annexin V/propidium iodide (PI) Kit (Invitrogen) was used. After appropriate staining, the cells were analyzed by the flow cytometry.

To confirm if KPC1 knockdown by RNA (Ad‐shKPC1) was involved in H9c2 cells apoptosis, 48 hr after transfection, the cells were exposed to specific treatment with H/R. Because Ad‐shKPC1 carried the specific GFP‐tag, the special Annexin V/TRITC Kit (Invitrogen) was used in flow cytometry analysis (Huang et al., 2011).

### 2.11 Bax protein stability assay

Forty‐eight hours after transfection, the cells were exposed to specific treatment with H/R. After treated with cycloheximide (CHX, 10 μg/ml) for 0–6 hr or MG132 (10 μM) for 0–8 hr, the cells were harvested for western blot analysis.

### 2.12 Mitochondrial membrane potential detection

Following transfection and H/R treatment, the mitochondrial membrane potential (MMP) was measured using a MitoProbe JC‐1 Assay Kit (M34152, Life). H9c2 cells were incubated with JC‐1 (1 μM each well) for 20 min. After washing, the cells were observed and photographed. The ratios of red‐to‐green fluorescence were quantified to evaluate the degree of damage to the mitochondrial membrane.

Because Ad‐shKPC1 carried the specific GFP‐tag, only the intensity of red fluorescence in Ad‐shKPC1 or Ad‐shCtrl treatment group was quantitated to estimate the degree of mitochondrial membrane damage.

### 2.13 Bax mitochondrial translocation assay

After transfection and H/R treatment, the slides were incubated with MitoTracker(M7512, invitrogen) and anti‐Bax antibody (1:200, #2772, CST). Then, cells were incubated with secondary antibody (1:500, CST) conjugated with fluorescein isothiocyanate (FITC). Using DAPI to label cell nucleus, the slides were observed and photographed.

### 2.14 qPCR

Total RNAs were extracted from H9c2 cells with a Trizol Reagent (Invitrogen). Complementary DNAs (cDNAs) were synthesized with a RevertAid First Strand cDNA Synthesis Kit (Thermo Fisher Scientific). Quantitative real‐time polymerase chain reaction (qRT‐PCR) analyses of individual cDNA were performed with a FastStart Universal SYBR Green Master (Roche) with a Real‐time PCR System (ABI‐7000) as described previously (Silva et al., 2012). The primer sequences were: KPC1 (5′–3′): CTGCGTCCAATAAGTCCAGC (forward), GACGTCATCTTTCACCGCTC (reverse).

### 2.15 Co‐immunoprecipitation (Co‐IP)

To examine the interaction between Bax and KPC1, the cells were lyzed with RIPA buffer (Millipore). After centrifugation, the supernatant was incubated with anti‐KPC1 antibody (sc‐101122, Santa Cruz) at 4°C overnight. The subsequent co‐immunoprecipitation (Co‐IP) was performed with a Pierce Co‐Immunoprecipitation Kit (26149, Thermo Fisher Scientific) following the manufacturer's instructions. After the final elution, the samples were collected for western blot analysis (Tang et al., 2018).

### 2.16 Statistical analysis

Statistical analysis was performed with PASW Statistics 18 (SPSS). All data were evaluated with two‐tailed, unpaired Student's *t* tests or one‐way analysis of variances (ANOVAs) followed by *t* tests and are expressed as the mean ± *SD*. The *p*‐values < .05 were considered significant.

## 3 RESULTS

### 3.1 KPC1 expressions in CHD hearts, infarcted hearts, I/R hearts, and H/R H9c2 cells

**Figure 1:**
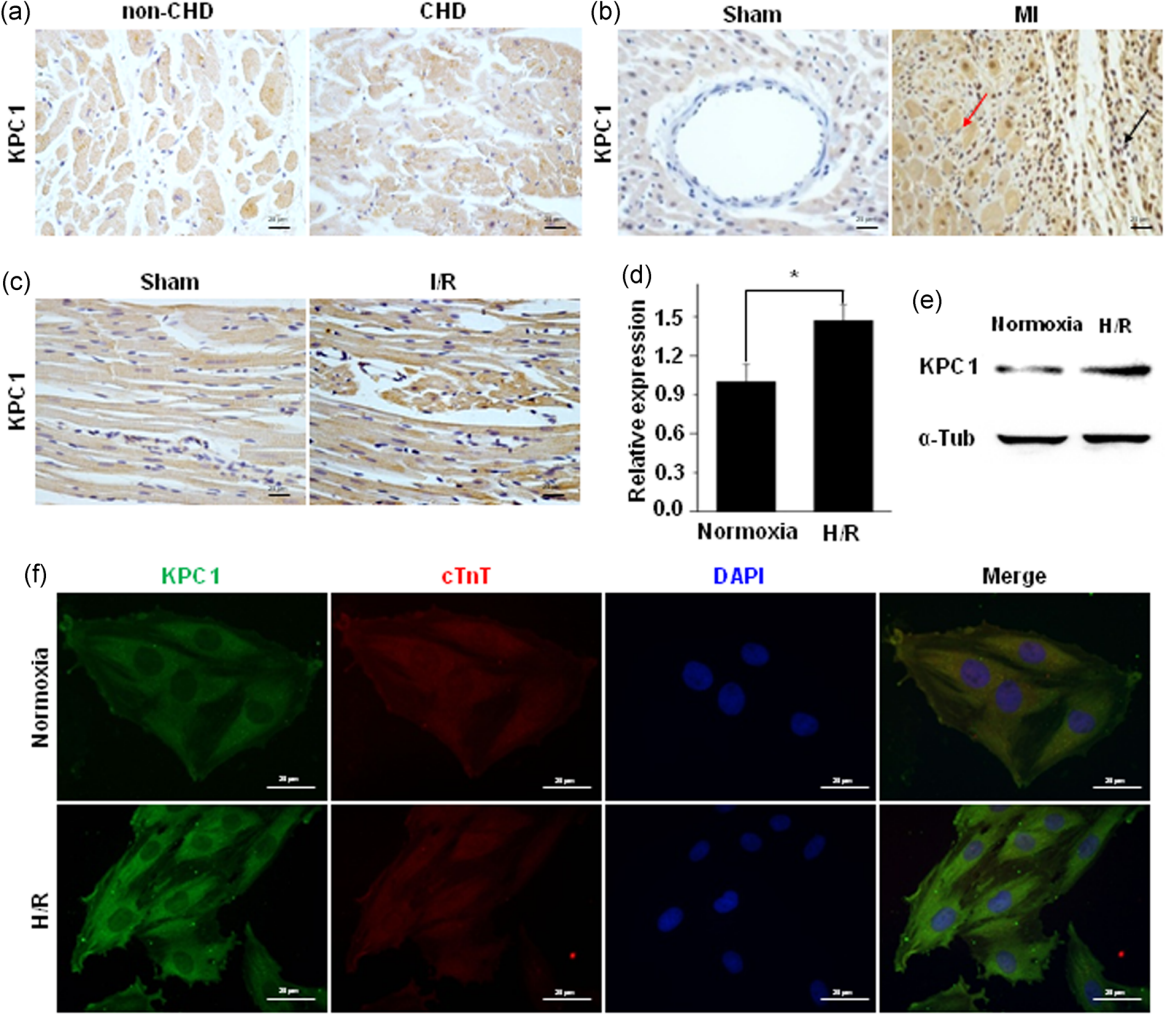
KPC1 expressions in CHD hearts, infarcted hearts, I/R hearts, and H/R H9c2 cells. (a) KPC1 expression level was increased in CHD hearts compared with non‐CHD hearts, *n* = 3. (b) KPC1 showed the increased expressions in infarcted area (black arrow) and in peri‐infarcted (red arrow) area of myocardium infarction, *n* = 6. (c) KPC1 expressions in the sham group and the I/R injury group were detected by IHC staining, *n* = 5. (d) Increased mRNA of KPC1 in myocardium infarction was detected by the qPCR, *p < .05, *n* = 3. (e) The increased KPC1 expression level was found in H/R H9c2 cells, *n* = 3. (f) Double immunostaining of KPC1 and c‐TnT showed the increased expression of KPC1 in H/R H9c2 cells compared with the normoxia group, *n* = 3. CHD, coronary heart disease; H/R, hypoxia and reoxygenation; IHC, immunohistochemistry; I/R, ischemia‐reperfusion [Color figure can be viewed at wileyonlinelibrary.com]

To explore the expression of KPC1 in CHD and in infarcted hearts, immunohistochemical staining was used to detect it in CHD and in I/R hearts. As shown in Figure 1a–c, KPC1 expression was increased in the CHD hearts, in ischemic, and infarcted areas of hearts (quantitative analysis results were shown in Figure S1a–c). Subsequently, to mimic the increased KPC1 expression in I/R hearts in vivo, KPC1 mRNA and protein expressions in the H/R H9c2 cells in vitro were detected by qPCR and western blot analysis, respectively. As shown in Figure 1d,e the levels of both KPC1 mRNA and proteins were markedly increased when exposed to H/R (quantitative analysis results were shown in Figure S1d). Then, to further assess if KPC1 expression was mainly located within cardiomyocytes, double staining of KPC1 and cTnT were performed and as shown in Figure 1f, KPC1 expression was obviously increased in cardiomyocytes. These results suggested that KPC1 expression was induced in cardiomyocytes following ischemia.

### 3.2 Knockdown of KPC1 worsens H/R‐driven H9c2 cell apoptosis

**Figure 2:**
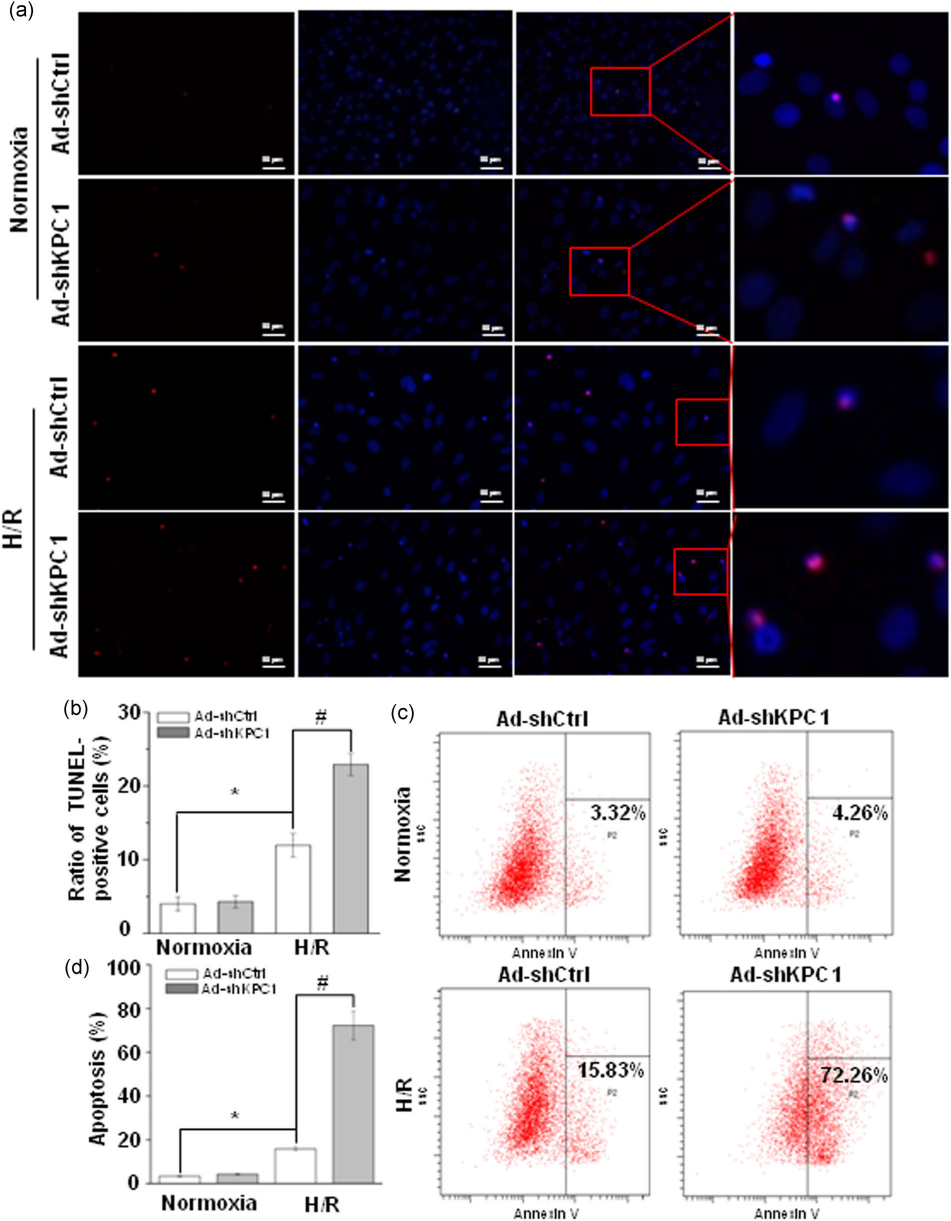
Knockdown of KPC1 worsens H/R‐driven H9c2 cell apoptosis. (a) Representative images of TUNEL staining of H9c2 cells treated with normoxia or H/R, *n* = 3. (b) Ratio of TUNEL‐positive H9c2 cells was analyzed, *p < .05, ^#^ *p* < .001, *n* = 6. (c), (d) KPC1 knockdown significantly accelerated H9c2 cell apoptosis after H/R, *p < .05, ^#^ *p* < .001, *n* = 3. H/R, hypoxia and reoxygenation; TUNEL, terminal‐deoxynucleotidyl transferase‐mediated nick end labeling [Color figure can be viewed at wileyonlinelibrary.com]

To observe the role of KPC1 in H/R‐induced apoptosis of H9c2 cells, loss‐of‐function of KPC1 was achieved by knockdown of KPC1 by shRNA (Ad‐shKPC1). The TUNEL staining was used to evaluate H9c2 cell apoptosis in H/R conditions. As shown in Figure 2a,b, the positive cells with typical red fluorescence indicated that apoptotic cells were increased in H/R‐treated H9c2 cells, and knockdown of KPC1 by shRNA exacerbated cell apoptosis driven by H/R. To further quantify cell apoptosis, flow cytometry assays of Annexin V/PI were used to confirm the specific effects, and as shown in Figure 2c,d, similar results were found. These results suggested that knockdown of KPC1 deteriorated the H9c2 cell apoptosis induced by H/R.

### 3.3 Forced KPC1 expression alleviates H/R H9c2 cell apoptosis

**Figure 3:**
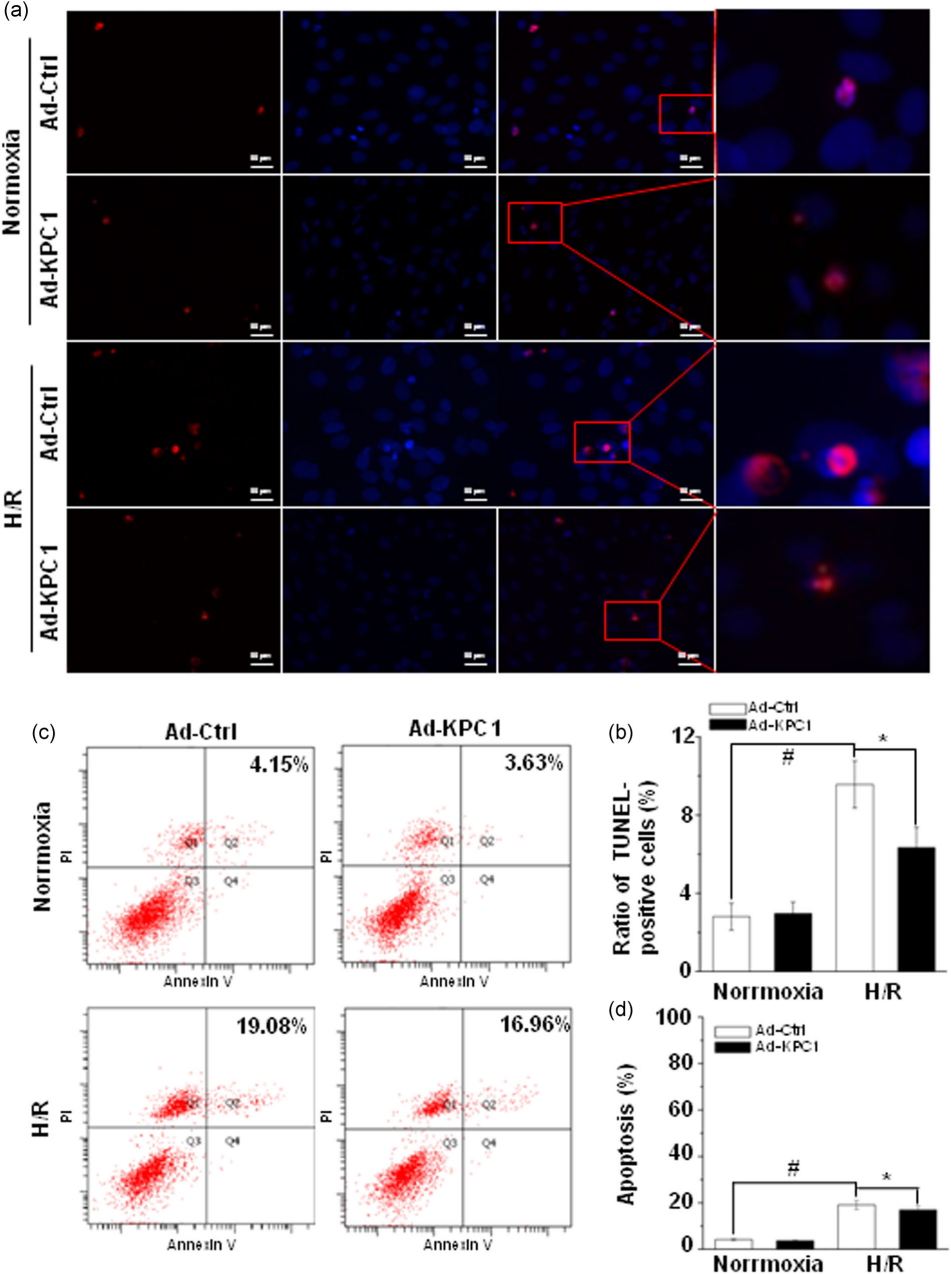
Forced KPC1 expression alleviates H/R‐driven H9c2 cell apoptosis. (a) Representative images of TUNEL staining of H9c2 cells treated with normoxia or H/R, *n* = 3. (b) Forced expression of KPC1 significantly decreased the ratio of H9c2 cell apoptosis, ^#^ *p* < .001, *p < .05, *n* = 6. (c), (d), Forced expression of KPC1 significantly alleviated H9c2 cell apoptosis after H/R, ^#^ *p* < .001, *p < .05, *n* = 3. H/R, hypoxia and reoxygenation; TUNEL, terminal‐deoxynucleotidyl transferase‐mediated nick end labeling [Color figure can be viewed at wileyonlinelibrary.com]

To further confirm the role of KPC1 in H/R‐induced apoptosis of H9c2 cells, gain‐of‐function of KPC1 was performed by the forced KPC1 expression by adenovirus (Ad‐KPC1). The TUNEL staining showed that Ad‐KPC1 clearly reduced H9c2 cell apoptosis by H/R as shown in Figure 3a,b. Simultaneously, flow cytometry assays showed similar results in Figure 3c,d. These data suggested that overexpression of KPC1 had protective effects on H/R‐induced H9c2 cell injury.

### 3.4 KPC1 improved mitochondrial function in H/R H9c2 cells

**Figure 4:**
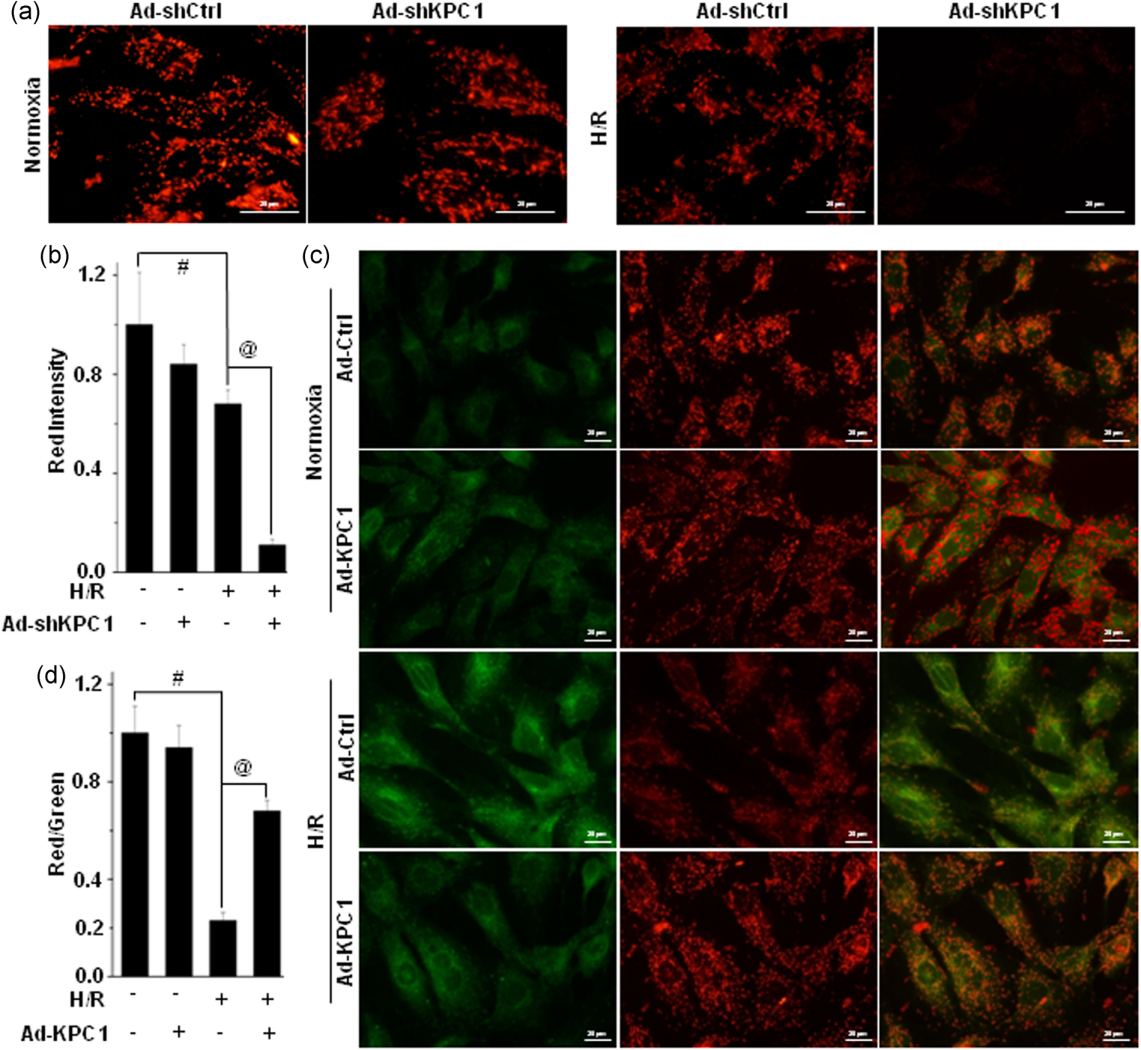
KPC1 involves in the regulation of mitochondrial function in H/R H9c2 cells. MMP was detected by JC‐1 fluorescence staining (red: normal; green: apoptosis). (a) Typical images of JC‐1 fluorescence staining in H/R H9c2 cells pretreated with or without KPC1 knockdown by shRNA. (b) Quantitative analysis of red intensity in (a), ^#^ *p* < .001, ^@^ *p* < .001, *n* = 6. (c), (d) The forced expressions of KPC1 improved mitochondrial function in H/R H9c2 cells. (c) Typical images for H/R H9c2 cells pretreated with or without Ad‐KPC1 of JC‐1 fluorescence staining. (d) Quantitative analysis of membrane potential shown in (c), ^#^ *p* < .001, ^@^ *p* < .001, *n* = 6. H/R, hypoxia and reoxygenation; MMP, mitochondrial membrane potential; shRNA, short‐hairpin RNA [Color figure can be viewed at wileyonlinelibrary.com]

MMP (∆*ψ*_m_) is a key indicator of mitochondrial function, and mitochondrial aggregation is before cytochrome *c* release from mitochondria during apoptosis (Huang et al., 2011). To first assess if KPC1 is involved in the changes in ∆*ψ*_m_ exposed to H/R, JC‐1 staining was performed for the traits of either monomer in the cytoplasm with green fluorescence or aggregated in mitochondria with red fluorescence. As shown in Figure 4a, H/R notably decreased the degree of red fluorescence intensity compared with normoxic conditions, indicating loss of mitochondrial membrane potential. KPC1 knockdown remarkably reduced the mitochondrial membrane potential with lower red fluorescence intensity. In contrast, the specific effects could be abolished by forced KPC1 expression, which was characterized by a higher ratio of red/green fluorescence shown in Figure 4c. Thus, KPC1 protected H/R‐induced H9c2 cell apoptosis through restoring mitochondria**l function.**

### 3.5 KPC1 involves in the regulation of cytochrome *c* release

**Figure 5:**
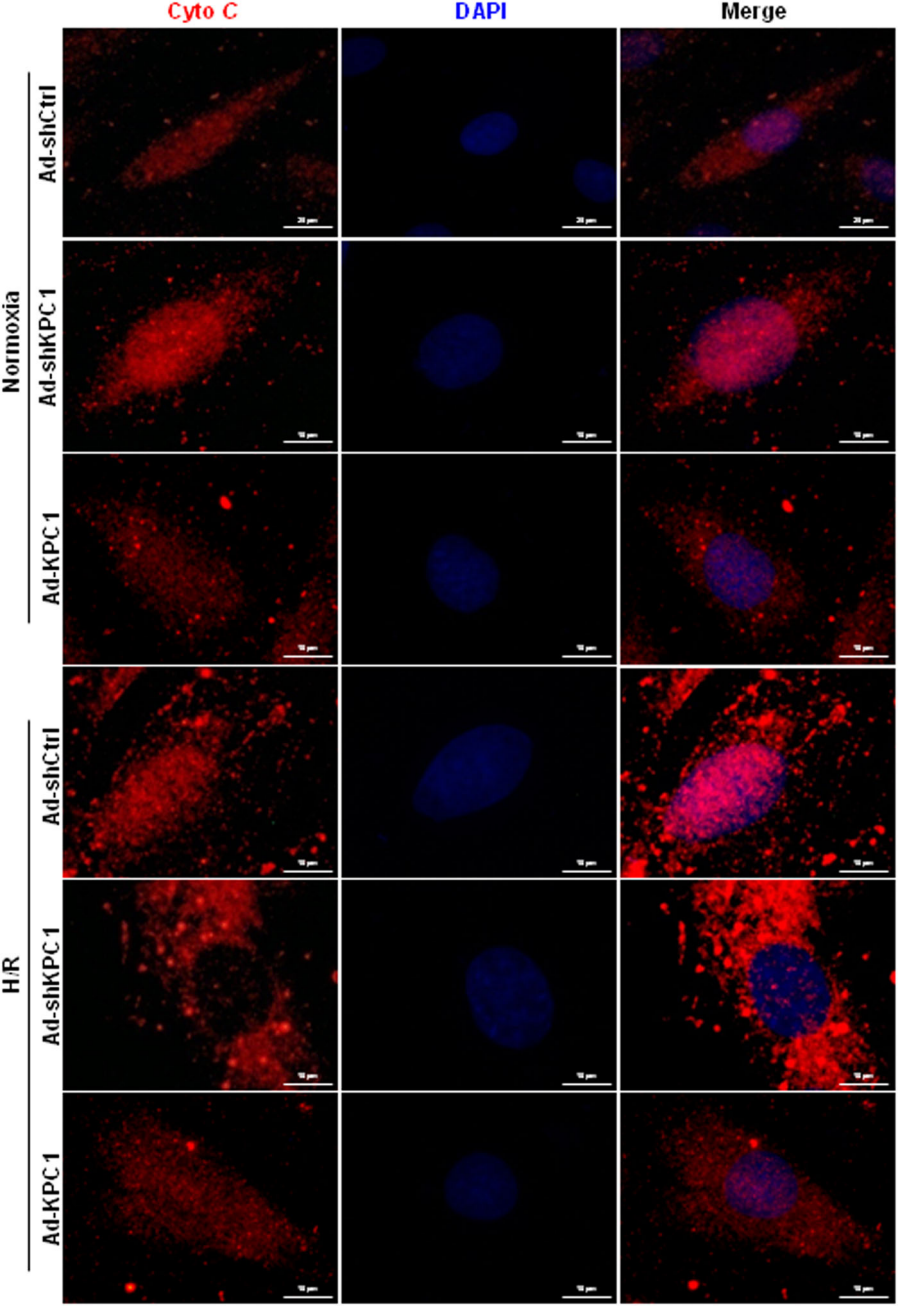
KPC1 decreases cytochrome *c* release from mitochondria. Cytochrome *c* releases from mitochondria were analyzed by double fluorescence staining of cytochrome *c* and DAPI. Under normoxic conditions, KPC1 knockdown slightly increased cytochrome *c* release from mitochondria in H9c2 cells. When exposed to H/R, the cells showed increased cytochrome *c* release, KPC1 knockdown further accelerated cytochrome *c* release. The specific effects could obviously be reversed by the overexpression of KPC1, *n* = 3. DAPI, 4',6‐diamidino‐2‐phenylindole; H/R, hypoxia and reoxygenation [Color figure can be viewed at wileyonlinelibrary.com]

Cytochrome *c* release from mitochondria to cytosol and activation of the caspase cascades after apoptotic stimuli, leading to cell death (Huang et al., 2011; Zhang et al., 2013). After finding the protective effects of KPC1 on H/R‐induced H9c2 cell apoptosis through improving mitochondrial function, we further evaluated the effects of KPC1 on cytochrome *c* release. As shown in Figure 5, H/R triggered cytochrome *c* release to cytoplasm by immunofluorescence staining. The specific effects could be obviously eliminated by overexpression of KPC1. Combined with the effects of KPC1 on mitochondrial membrane potential recovery, we speculated that KPC1 could prevent cytochrome *c* release from mitochondria through decreasing mitochondrial aggregation during apoptosis.

### 3.6 KPC1 regulates bax stability in H/R H9c2 cells through a proteasomal pathway

**Figure 6:**
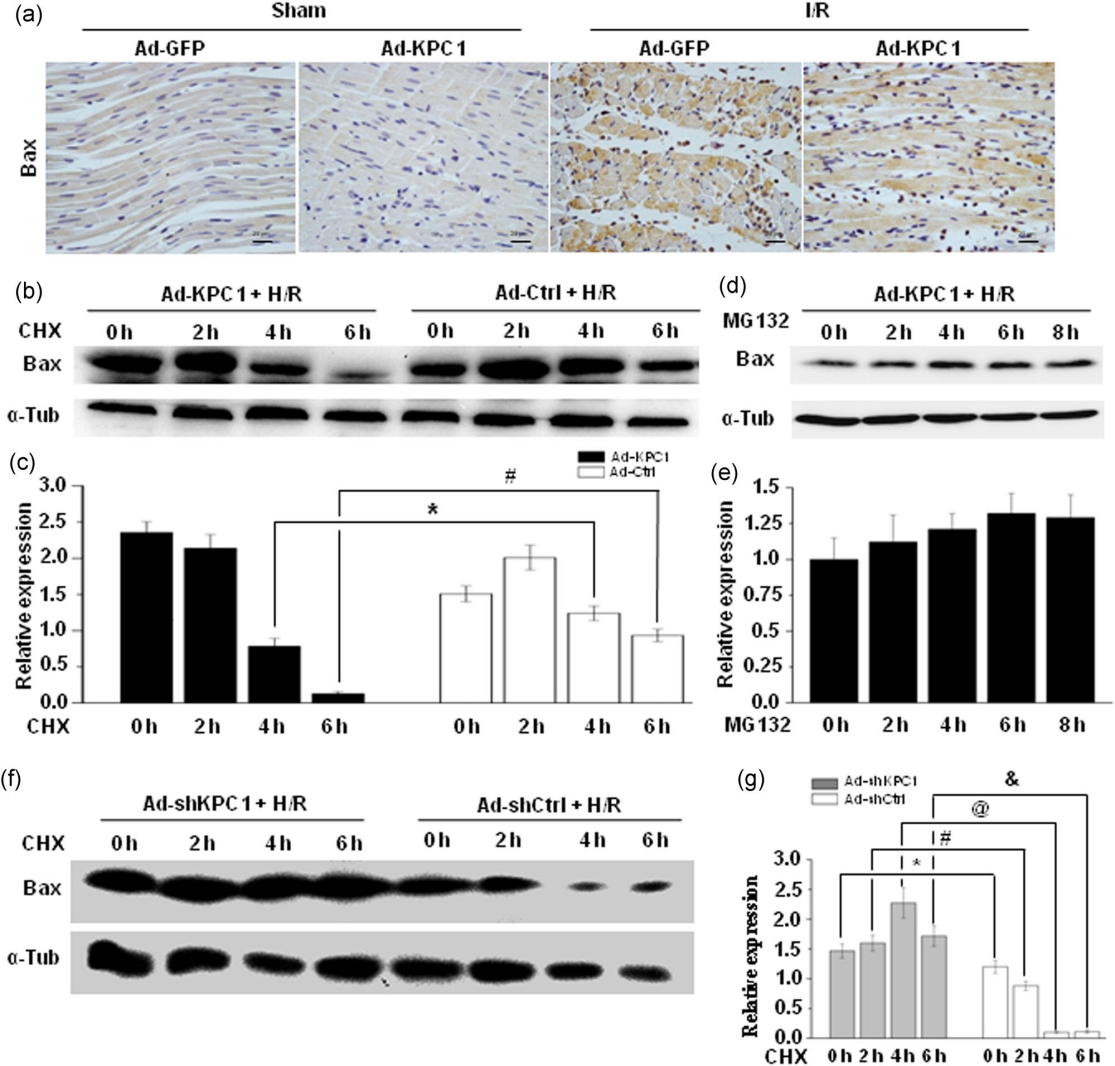
KPC1 regulates Bax protein stability in H/R H9c2 cells through a proteasomal pathway. (a) Bax expression was detected on different groups by immunostaining, *n* = 6. Forty‐eight hours after H9c2 cells were pretreated with Ad‐KPC1, Ad‐Ctrl, Ad‐shKPC1, or Ad‐shCtrl, the cells were exposed under H/R conditions, and 10 μg/ml CHX were added for the indicated time. (b), (c) Bax stability was decreased in H/R H9c2 cells pretreated with Ad‐KPC1. Bax expressions were normalized to α‐tubulin, *p < .05, ^#^ *p* < .001, *n* = 3. (d), (e) Bax degradation by proteasome pathway was abolished by MG132. Bax expressions were normalized to α‐tubulin. (f), (g) Bax stability was increased in H/R H9c2 cells pretreated with Ad‐shKPC1 and Bax intensities were normalized to α‐tubulin intensities, *p < .05, ^#^ *p* < .001, ^@^ *p* < .001, ^&^ *p* < .001, *n* = 3. CHX, cycloheximide; H/R, hypoxia and reoxygenation [Color figure can be viewed at wileyonlinelibrary.com]

The loss of ∆*ψ*m under stress was generally attributed to increased levels of Bax (Huang et al., 2011; Zhang et al., 2013). Traditionally, Bax protein levels were first analyzed because of greater Bax levels and lower cells survival (Lesnefsky et al., 2017; Ong et al., 2015). After Ad‐KPC1 was injected into myocardium sites in the left ventricle anterior wall for 1 week, adenovirus‐mediated KPC1 overexpression was verified by IHC staining (Figure S1f). To explore whether the above effects were related to Bax, the Bax expression was detected in I/R myocardial tissues using IHC staining. As shown in Figure 6a, I/R heart tissues, following pretreatment of Ad‐GFP, revealed stronger expression of Bax compared with the sham groups (quantitative analysis results shown in Figure S1e). Of interest, heart pretreatment with Ad‐KPC1 remarkably reduced Bax levels triggered by I/R injury.

To reveal the underlying mechanism, Bax expression was detected in KPC1 gain‐of‐function and loss‐of‐function H/R‐induced H9c2 cells. Here, forced KPC1 expression markedly decreased Bax levels through accelerating Bax protein degradation as demonstrated in Figure 6b,c. Inversely, knockdown of KPC1 by shRNA remarkably delayed Bax protein degradation (Figure 6f,g). Furthermore, Bax protein degradation by KPC1 was obviously abolished by proteasomal inhibitor MG132 (Figure 6d,e) suggesting that KPC1 participated in the regulation of Bax proteasomal degradation.

### 3.7 The forced expression of KPC1 decreases bax mitochondria translocation in H/R H9c2 cells

**Figure 7:**
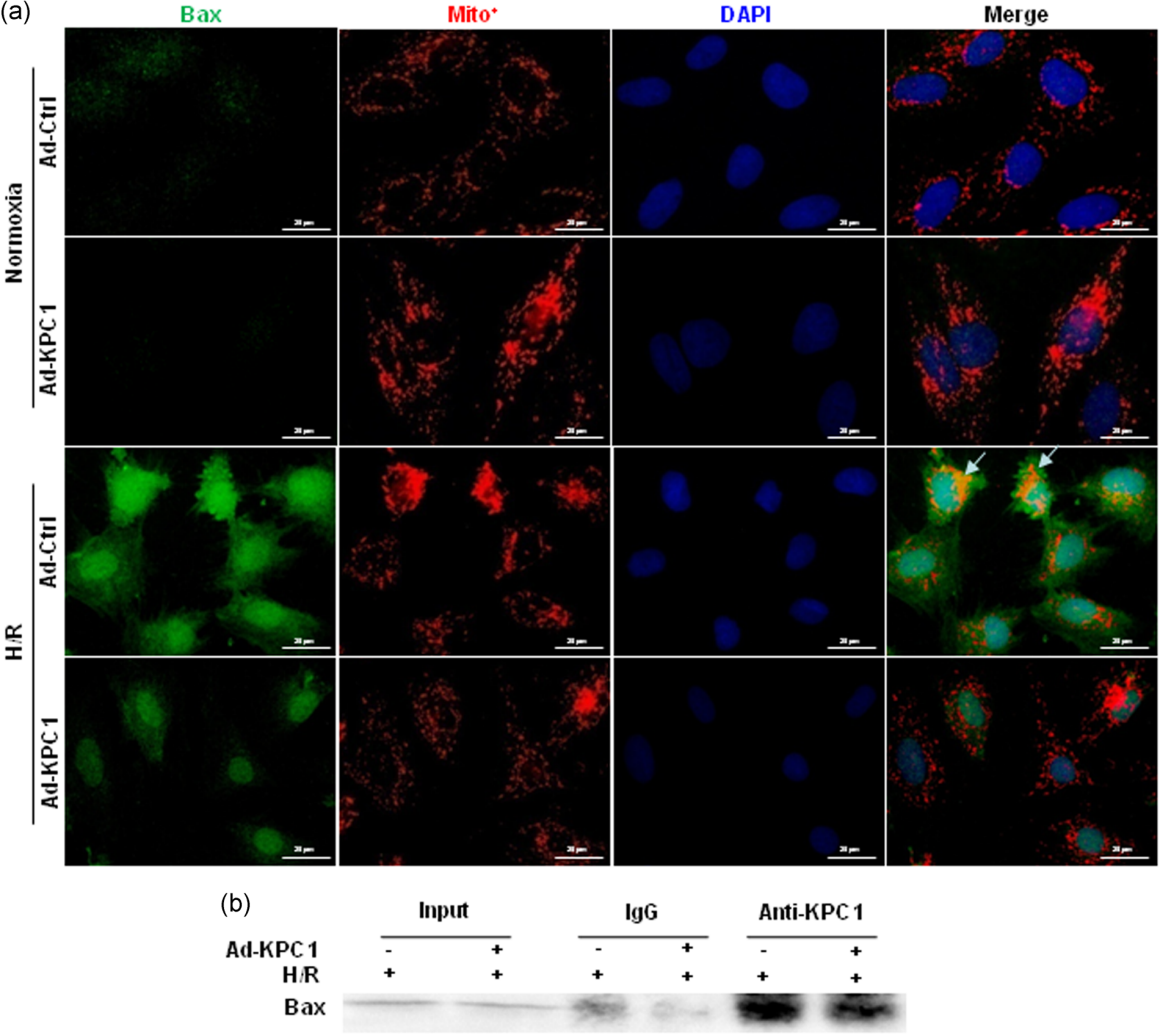
KPC1 interacts with endogenous Bax and decreases translocation of Bax to mitochondria in H/R H9c2 cells. (a) Two days after treated with Ad‐KPC1 or Ad‐Ctrl, the cells were exposed under H/R conditions. Double fluorescence staining of Bax and Mitotracker were analyzed in H/R cells pretreated with or without Ad‐KPC1. KPC1 decreases Bax translocation to mitochondria from cytoplasm (arrow). (b) Two days after H9c2 cells treated with Ad‐KPC1 or Ad‐Ctrl, the cells were exposed under H/R conditions. The cell lysate were immunoprecipitated with anti‐KPC1 and immunoblotted with anti‐Bax. H/R, hypoxia and reoxygenation [Color figure can be viewed at wileyonlinelibrary.com]

Really, Bax, functioning as a regulator of mitochondrial activity, deeply depends on translocation of Bax to the mitochondria besides considering Bax protein levels (Gross et al., 1998). We further evaluated if KPC1 was involved in Bax translocation to mitochondria in H/R H9c2 cells. As shown in Figure 7, Bax showed greater translocation to mitochondria in H/R H9c2 cells compared with normoxic cells. The forced KPC1 expressions did not obviously alter Bax levels or location in mitochondria under normoxic conditions (Figure 7a) but distinctly reversed the H/R‐induced Bax mitochondria translocation, demonstrating that KPC1 provides substantive protection against mitochondria‐dependent apoptotic action through targeting Bax.

### 3.8 KPC1 interacts with endogenous bax during apoptosis

To uncover if KPC1 directly mediated Bax function, Co‐IP of KPC1, and Bax were performed. As shown in Figure 7b, immunoprecipitation of KPC1 pulled down Bax, indicating that KPC1 interacted with endogenous Bax.

### 3.9 Overexpressing KPC1 decreases bax levels and cell apoptosis in I/R hearts

**Figure 8:**
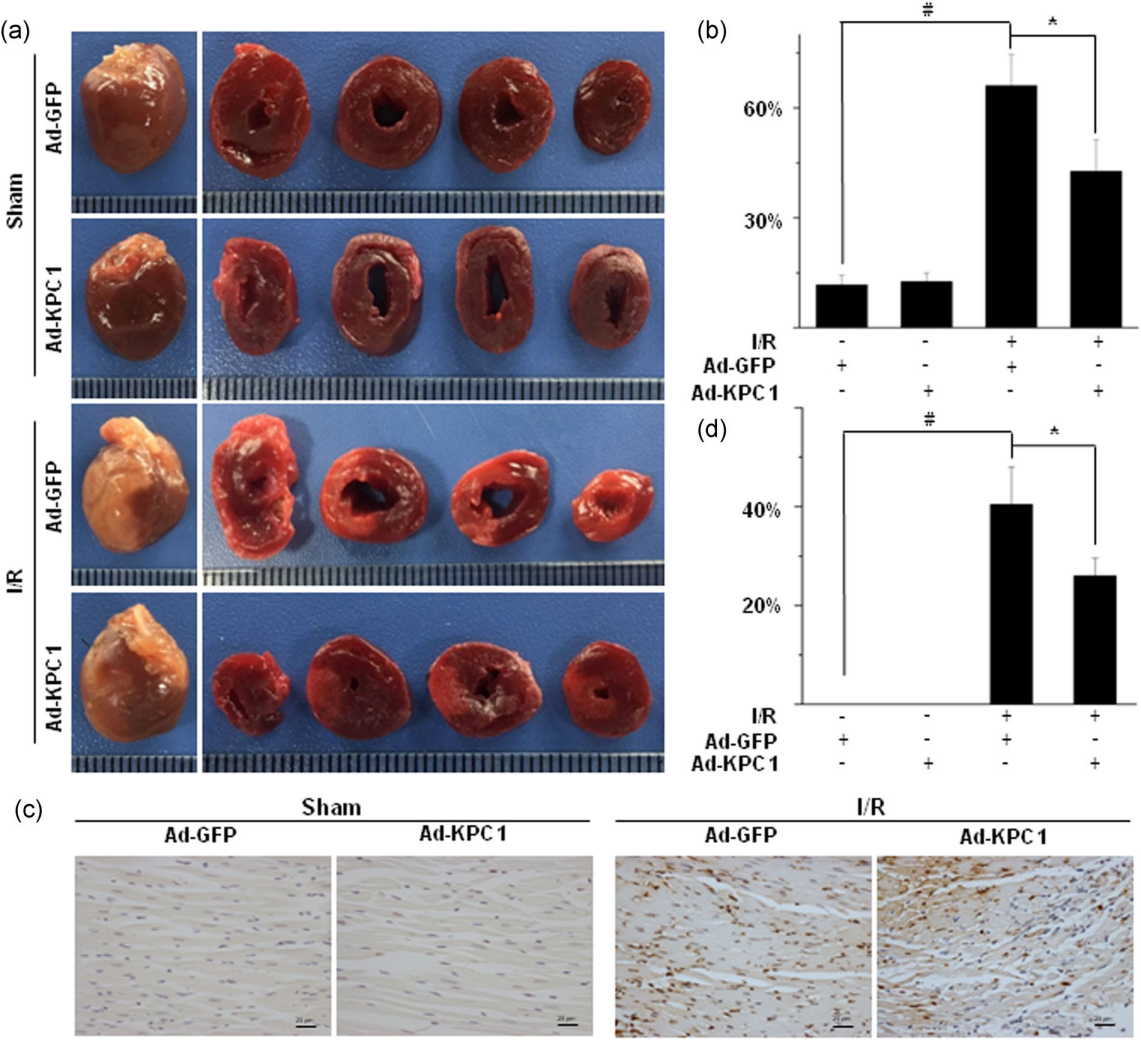
Overexpressing KPC1 decreases Bax expression and cell apoptosis in I/R hearts. (a), (b) Infarcted size was measured by the TTC staining. After I/R injury, ischemic area was easily observed compared with sham group. However, the infarcted sizes were reduced in Ad‐KPC1 pretreatment compared with Ad‐GFP pretreatment after I/R injury, ^#^ *p* < .001, *P < 0.05, *n* = 5. (c), (d) Apoptosis was detected on different groups by the TUNEL staining. The proportion of TUNEL‐positive cells in Ad‐KPC1 transfection group was significantly decreased after I/R injury compared with Ad‐GFP pretreatment and the I/R group, ^#^ *p* < .001, *p < .05, *n* = 5. GFP, green fluorescent protein; I/R, ischemia‐reperfusion; TTC, 5‐triphenyltetrazolium chloride; TUNEL, terminal‐deoxynucleotidyl transferase‐mediated nick end labeling [Color figure can be viewed at wileyonlinelibrary.com]

To further confirm the role of KPC1 in Bax levels and cell apoptosis in I/R hearts, an I/R heart injury model in rats was used. As a typical method, the TTC staining was used to evaluate I/R heart injury and infarcted size. Generally, TTC staining is brick‐red, which represents nonischemic myocardium, whereas white staining indicates infarcted area. As shown in Figure 8a,b, heart tissues with pretreatment with Ad‐GFP or Ad‐KPC1 did not show the changes in ischemic myocardium following the sham operation, indicating that Ad‐GFP and especially Ad‐KPC1 injection, did not cause heart damage. I/R myocardial tissues with pretreatment with Ad‐GFP showed more than 60% white area in left ventricle, demonstrating I/R. In contrast, I/R myocardial tissues with pretreatment with Ad‐KPC1 displayed less than 40% area in the left ventricle, illustrating that forced expression of KPC1 could contribute to cardioprotective effects.

To further affirm the beneficial effects of KPC1 in the I/R heart, cell apoptosis in heart tissues was investigated using the TUNEL staining. As shown in Figure 8c,d, I/R heart tissues with pretreatment with Ad‐GFP showed an almost 40% apoptotic ratio. In contrast, overexpression of KPC1 in I/R myocardial tissues markedly reduced the apoptotic ratio of cells in the left ventricle, which approached 25%, indicating that the pretreatment with Ad‐KPC1 decreased infarcted size by reducing cell apoptosis.

Combining in vitro and in vivo results, KPC1 regulated cell apoptosis in I/R hearts through controlling Bax function.

## 4 DISCUSSION

The results of this study show that KPC1 was identified as a novel factor protecting cardiomyocytes from apoptosis during H/R stress in vitro and I/R injury in vivo. The role of KPC1 in protecting cells from H/R‐ and I/R‐induced cell apoptosis is supported by several lines of evidence. First, cardiomyocytes showed increased KPC1 expression during H/R or I/R‐caused injury. Second, KPC1 knockdown exacerbated H/R‐induced H9c2 apoptosis, whereas KPC1 overexpression alleviated the damage. Finally, targeting Bax, KPC1 provided crucial protection against mitochondria‐dependent cell apoptosis (Figure S2).

When proteasomal degradation fails to satisfy the need to remove substrate proteins, PFI can occur at a specific location within the cell (Wang & Robbins, 2013). PFI frequently occurs in heart disease, especially in myocardial I/R injury (Li et al., 2011; Tian et al., 2012). Herein, we found that KPC1 expression was increased in cardiomyocytes following I/R injury in vivo and H/R in vitro. Knockdown of KPC1 exacerbated H/R‐driven H9c2 cell apoptosis, indicating that elevated KPC1 expression in ischemic hearts, especially in injured cardiomyocytes, could be an adaptative adjustment to the demand for enhancive turnover of structural and functional proteins. Indeed, KPC1 overexpression decreased cell apoptosis driven by H/R in vitro and I/R injury in vivo.

Forced KPC1 expression enhanced proteasomal function to degrade Bax, possibly through ubiquitination. Indeed, it was reported that Bax ubiquitination participated in apoptotic cell death (Cakir et al., 2017). However, literature has emerged that provides disputable findings about the E3 ligase responsibilities. Two studies have shown that the E3 ligase, parkin, can ubiquitinate the Bax BH3 domain, therefore, before transferring to mitochondria, cytoplasmic Bax was degraded through proteasome. (Charan, Johnson, Zaganelli, Nardozzi, & Lavoie, 2014; Johnson, Berger, Cortese, & Lavoie, 2012). Of interest, a recent study provided controversial evidence that parkin promotes dysregulated Bax proteasomal degradation in the mitochondria, causing essential protection against apoptotic activeness of Bax (Cakir et al., 2017; Lindenboim, Ferrandomay, Borner, & Stein, 2013). Our results showed that KPC1 functioned similarly to the parkin E3 ligase in the proteasomal degradation of Bax in cytoplasm with less translocation to mitochondria, resulting in recovery of mitochondrial function and reduction in cell apoptosis induced by I/R or H/R injury. Although a complete understanding of KPC1 function in protecting I/R and H/R‐induced cell apoptosis through Bax requires extensive future study, our studies suggest that KPC1 may serve as a potential target for treating cardiovascular diseases characterized by PFI, especially I/R‐injured myocardium.

NF‐κB (p50/p65) and p53 have been shown to activate Bax in I/R‐injured hearts (Liu, Xu, Cavalieri & Hock, 2006; Zhang et al., 2018). Interestingly, p53 is not only a direct transcriptional activator of Bax (Miyashita & Reed, 1995), but also activates KPC1 expression (Zhao et al., 2013). Recent studies showed that KPC1 ubiquitinates p105, leading to enhanced production of p50 but decreased levels of p65, which elicits suppression of tumor growth (Kravtsova‐Ivantsiv & Ciechanover, 2015; Kravtsova‐Ivantsiv et al., 2015). Of note, knockdown of NF‐κB p65 expression or inhibition of its activity lessens cell apoptosis through reducing Bax expression (Li et al., 2013; Qu, Zhang, & Wu, 2015). We found that KPC1 accelerated proteasomal degradation of Bax through forming a complex of KPC1‐Bax. Therefore, p53, KPC1, and NF‐κB likely form an integrated system to regulate Bax activity during I/R or H/R‐induced cardiomyocyte apoptosis.

Taken together results, our study demonstrated that KPC1 exerted protective effects on I/R‐ and H/R‐induced cardiomyocyte apoptosis through accelerating proteasomal degradation of Bax and reducing Bax mitochondria translocation.

## CONFLICT OF INTERESTS

The authors declare that they have no conflict of interests.

## AUTHOR CONTRIBUTIONS

Ye Yuan performed main cells experimental and drafted the manuscript. Yong‐yi Wang carried out cells experimental and drafted the manuscript. Xin Liu carried out qPCR. Bin Luo carried out data evaluation. Lei Zhang and Fei Zheng participated in protein assay. Xing‐Yuan Li participated in the immunostaining. Ling‐Yun Guo carried out flow cytometry. Lu Wang participated in TUNEL analysis. Miao Jiang, Yu‐wen Yan, and Jian‐ye Yang performed the analysis. Shi‐You Chen and Jia‐Ning Wang participated in the design of the study and performed the statistical analysis. Jun‐Ming Tang conceived of the study, and participated in its design and coordination and helped to draft the manuscript. All authors read and approved the final manuscript.

## DATA AVAILABILITY STATEMENT

The data are openly available in the Open Repository of National Natural Science Foundation of China (NSFC‐OR).

## DECLARATIONS

### Ethics approval and consent to participate

The study was approved by the Institutional Review Board of Hubei University of Medicine. All animals received care according to the Principles of Laboratory Animal Care formulated by the National Society for Medical Research and the Guide for the Care and Use of Laboratory Animals. Animal surgical procedures were approved by the Care of Experimental Animals Committee of Hubei University of Medicine.

## Supporting information

Supplementary information

Supplementary information

Supplementary information
