# Supplementary figures and images for: KPC1 alleviates hypoxia/reoxygenation‐induced apoptosis in rat cardiomyocyte cells though BAX degradation
Source: J Cell Physiol. 2019 May 30;234(12):22921–34. doi: 10.1002/jcp.28854 (PMC6771896; doi:10.1002/jcp.28854)

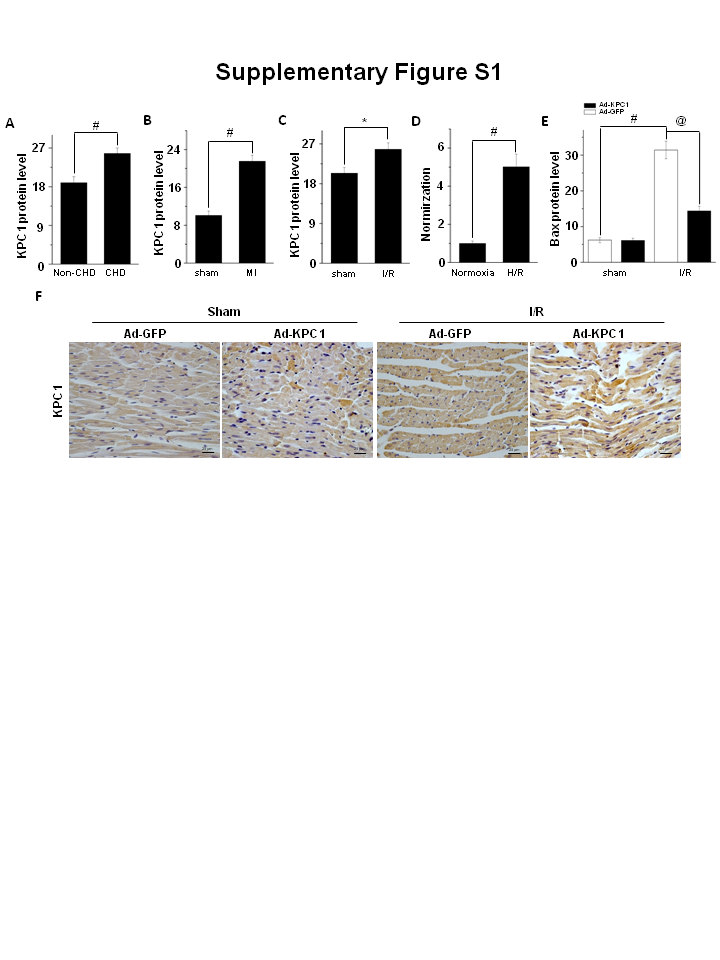

Supplement: Supplementary file 1 — Supplementary information [file JCP-234-22921-s001.TIF]

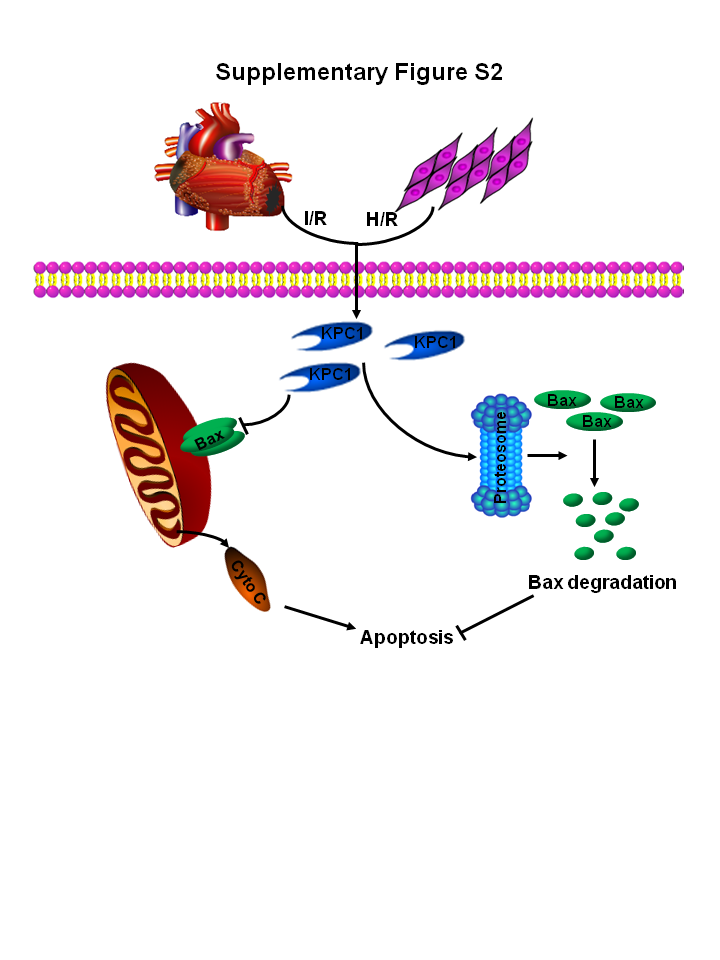

Supplement: Supplementary file 2 — Supplementary information [file JCP-234-22921-s002.TIF]
