# Supplementary material for: KPC1 alleviates hypoxia/reoxygenation‐induced apoptosis in rat cardiomyocyte cells though BAX degradation
Source: J Cell Physiol. 2019 May 30;234(12):22921–34. doi: 10.1002/jcp.28854 (PMC6771896; doi:10.1002/jcp.28854)
Supplement: Supplementary file 3 — Supplementary information [file JCP-234-22921-s003.doc]

**KPC1 alleviates hypoxia/reoxygenation-induced apoptosis in rat cardiomyocyte cells though BAX degradation**

Ye Yuan^1,4*^, Yong-yi Wang^2*^, Xin Liu^5^, Bin Luo^3^, Lei Zhang^1,4^, Fei Zheng^1^, Xing-Yuan Li^1^, Ling-Yun Guo^1^, Lu Wang^1^, Miao Jiang^1^, Ya-mu Pan^1^, Yu-wen Yan^1^, Jian-ye Yang^1^, Shi-You Chen^6^, Jia-Ning Wang^1,4^, Jun-Ming Tang^1,3,4#^

^1^Institute of Clinical Medicine and Department of Cardiology, Renmin Hospital, Hubei University of Medicine, Shiyan, Hubei 442000, P. R. China;

^2^Department of Cardiovascular Surgery, Ren Ji Hospital, School of Medicine, Shanghai Jiao Tong University, Shanghai, P. R. China;

^3^Department of Physiology, School of Basic Medicine Science, Hubei University of Medicine, Hubei, 442000, P. R. China.

^4^Institute of Biomedicine and Key Lab of Human Embryonic Stem Cell of Hubei Province, Hubei University of Medicine, Hubei 442000, P. R. China;

^5^Laboratory Animal Center, Hubei University of Medicine, Hubei442000, P. R. China.

^6^Department of Physiology & Pharmacology, The University of Georgia, Athens, GA 30602, USA.

*Co-first author

^#^Corresponding author: Jun-Ming Tang, MD, PhD

Tel.:+86-719/8637706; Fax: 86-719/8637792;

E-mail: [tangjm416@163.com](mailto:tangjm416@163.com)

**Supplementary Figure S1 Legends**

A. Quantification of KPC1 expression shown in Figure 1A, #*P* <0.001, n=3. B. Quantification of KPC1 expression shown in Figure 1B, ^#^*P*<0.001, n=5. C. Quantification of KPC1 expression shown in Figure 1C, **P* < 0.05, n=6. D. Quantification of KPC1 expression shown in Figure 1E by normalized to α-tubulin, #*P* < 0.001, n=3. E. Quantification of Bax expression shown in Figure 6A, **P* < 0.001, @*P* < 0.001, n=5. F. KPC1 expression was detected by IHC immunostaining on different groups, n=6.

**Supplementary Figure S2 Legends**

KPC1 expression was increased in left ventricular cardiomyocytes of patients with coronary heart disease (CHD), in I/R-myocardium in vivo and in hypoxia and reoxygenation (H/R)-induced cardiomyocytes in vitro. Overexpression of KPC1 reduced cytochrome c release from mitochondria and prevented Bax translocation to mitochondria. Taken together, KPC1 exerted protective effects on I/R- and H/R-induced cardiomyocyte apoptosis through accelerating proteasomal degradation of Bax and reducing Bax mitochondria translocation.
